# Current Trends in Experimental and Computational Approaches to Combat Antimicrobial Resistance

**DOI:** 10.3389/fgene.2020.563975

**Published:** 2020-11-06

**Authors:** Madangchanok Imchen, Jamseel Moopantakath, Ranjith Kumavath, Debmalya Barh, Sandeep Tiwari, Preetam Ghosh, Vasco Azevedo

**Affiliations:** ^1^Department of Genomic Science, School of Biological Sciences, Central University of Kerala, Kasaragod, India; ^2^Centre for Genomics and Applied Gene Technology, Institute of Integrative Omics and Applied Biotechnology, Purba Medinipur, India; ^3^Laboratório de Genética Celular e Molecular, Departamento de Biologia Geral, Instituto de Ciências Biológicas, Universidade Federal de Minas Gerais, Belo Horizonte, Brazil; ^4^Department of Computer Science, Virginia Commonwealth University, Richmond, VA, United States

**Keywords:** antibiotic resistance, multidrug resistance, whole genome sequence, metagenomics, next generation sequencing, nanoparticles

## Abstract

A multitude of factors, such as drug misuse, lack of strong regulatory measures, improper sewage disposal, and low-quality medicine and medications, have been attributed to the emergence of drug resistant microbes. The emergence and outbreaks of multidrug resistance to last-line antibiotics has become quite common. This is further fueled by the slow rate of drug development and the lack of effective resistome surveillance systems. In this review, we provide insights into the recent advances made in computational approaches for the surveillance of antibiotic resistomes, as well as experimental formulation of combinatorial drugs. We explore the multiple roles of antibiotics in nature and the current status of combinatorial and adjuvant-based antibiotic treatments with nanoparticles, phytochemical, and other non-antibiotics based on synergetic effects. Furthermore, advancements in machine learning algorithms could also be applied to combat the spread of antibiotic resistance. Development of resistance to new antibiotics is quite rapid. Hence, we review the recent literature on discoveries of novel antibiotic resistant genes though shotgun and expression-based metagenomics. To decelerate the spread of antibiotic resistant genes, surveillance of the resistome is of utmost importance. Therefore, we discuss integrative applications of whole-genome sequencing and metagenomics together with machine learning models as a means for state-of-the-art surveillance of the antibiotic resistome. We further explore the interactions and negative effects between antibiotics and microbiomes upon drug administration.

## Introduction

**FIGURE 1:**
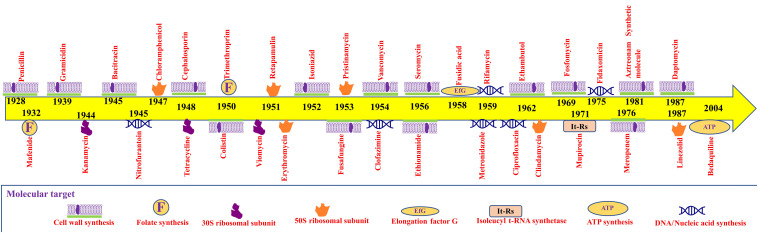
Timeline of antibiotics’ discoveries and their molecular targets.

Microorganisms are ubiquitous in nature and play crucial roles in the body’s biochemical functions. Despite their importance, opportunistic and pathogenic microbes have evolved to bypass the immune system. Gram-negative bacteria in particular are of clinical importance, as they produce metabolites, such as endotoxin and lipopolysaccharide, which are major causes of infection (Baron, 1996). Fleming’s serendipitous discovery of penicillin in 1928 began the antibiotic revolution and provided proof that natural compounds could selectively inhibit the growth of pathogens (Gaynes, 2017). Since then, antibiotics have been purified from various microbial, plant, and synthetic sources (Charest et al., 2005; de Lima Procópio et al., 2012; Khameneh et al., 2019). Today, antibiotics of various inhibitory mechanisms, such as inhibition of cell wall synthesis, protein synthesis, DNA replication, and folic acid synthesis, are widely available. The downside of such availability is the misuse of drugs and accelerated antibiotic resistance (Figure 1). Antibiotic resistance in bacterial pathogens is widespread. Approximately 2.8 million cases of new infection and about 35,000 deaths occur annually in the US alone (Tanzi, 2020). Antibiotic resistance is a global problem threatening the modern world (Table 1). According to the Global Antimicrobial Resistance Surveillance System report in 2017, the major clinically relevant antibiotic resistance species are *Escherichia coli*, *Klebsiella pneumoniae*, *Acinetobacter* spp., *Staphylococcus aureus*, *Salmonella* spp., *Shigella* spp., *Streptococcus pneumoniae*, and *Neisseria gonorrhoeae*. The spread of antibiotic resistance is also determined by geographical and climatic condition, policies, and socioeconomic status (Wellington et al., 2013). During organ transplantation, antibiotics play an indispensable role in combating nosocomial or secondary infection (Munita and Arias, 2016). The rise of antibiotic resistance had compelled researchers to discover novel antimicrobial agents and formulate polices to prevent disease through contact tracing and quarantine regimes (Roca et al., 2015). It also directly effects the economy through prolonged hospital stays, longer treatments, expensive medicines, and the involvement of manual labor in monitoring to avoid further progression. Antibiotic resistance is of special concern in Asian and African countries with weaker socioeconomic factors, including poor sanitation and general health (MacPherson et al., 2009; Collignon et al., 2018; Hendriksen et al., 2019b).

**TABLE 1:** Overview of Antibiotic resistance in *E. coli* toward major antibiotics in Australia, India, South Africa, the United Kingdom, and United States (Source: The Center for Disease, Dynamics Economics & Policy. ResistanceMap: Antibiotic resistance. 2020. https://resistancemap.cddep.org/AntibioticResistance.php. Date accessed: May 31, 2020).

Antibiotics	Year	Aminoglycosides		Aminopenicillins		Amoxicillin-clavulanate		Carbapenems		Cephalosporins (3rd gen)		Fluoroquinolones		Piperacillin-tazobactam		Glycylcyclines		Polymyxins	
										
		AMR (%)	Total sample tested	AMR (%)	Total sample tested	AMR (%)	Total sample tested	AMR (%)	Total sample tested	AMR (%)	Total sample tested	AMR (%)	Total sample tested	AMR (%)	Total sample tested	AMR (%)	Total sample tested	AMR (%)	Total sample tested
Australia	2013	8	2958	52	2958	–	–	0	2958	8	2958	10	2958	–	–	–	–	–	–
	2014	8	3493	52	3493	21	3493	0	3493	9	3493	10	3493	3	3493	–	–	–	–
	2015	8	3994	55	3992	22	3995	0	3993	11	3994	13	3994	3	3974	–	–	–	–
	2016	8	4353	54	4353	22	4354	0	4353	11	4355	12	4353	3	4345	–	–	–	–
	2017	9	4355	54	4353	22	4354	0	4353	11	4355	12	4353	6	4345	–	–	–	–
India	2008	61	62	–	–	–	–	9	55	71	63	83	63	36	61	–	–	–	–
	2009	75	165	–	–	–	–	5	155	82	165	90	165	30	162	–	–	–	–
	2010	70	196	–	–	–	–	5	185	77	195	88	196	28	194	–	–	–	–
	2011	66	309	–	–	–	–	7	281	79	281	89	310	30	298	–	–	–	–
	2012	70	485	–	–	–	–	13	412	82	472	85	486	41	471	2	180	3	118
	2013	63	458	–	–	–	–	11	445	80	441	85	458	34	442	0	205	1	186
	2014	61	422	–	–	–	–	11	408	83	400	84	418	37	407	3	144	3	186
	2015	26	3333	–	–	–	–	15	3108	78	3072	78	3316	36	2295	1	2242	0	2518
	2016	24	225	–	–	–	–	20	223	80	259	84	275	42	106	–	–	0	218
	2017	17	1619	–	–	–	–	18	1619	77	1619	84	1619	28	1619	–	–	1	1619
South Africa	2011	15	1726	81	1726	28	1726	0	1726	16	1726	28	1726	15	1726	–	–	–	–
	2012	19	3413	83	3416	29	1962	0	3380	19	3407	28	3407	15	3263	–	–	–	–
	2013	18	3814	79	2093	41	3835	0	3818	18	3766	27	3824	14	3803	–	–	–	–
	2014	19	4202	80	4199	29	2760	0	4159	19	4129	28	4205	16	4191	–	–	–	–
	2015	17	6526	84	3506	35	5951	0	6387	21	5633	29	5611	16	6107	–	–	–	–
	2016	17	6636	82	5825	34	6618	0	6489	23	6523	28	5804	16	6253	–	–	–	–
United Kingdom	2000	–	–	–	–	–	–	–	–	–	–	–	–	–	–	–	–	–	–
	2001	3	1355	51	1384	–	–	–	–	1	1154	6	1225	–	–	–	–	–	–
	2002	3	1906	52	1914	–	–	–	–	2	1703	7	1729	–	–	–	–	–	–
	2003	5	2195	55	2171	–	–	–	–	3	2018	11	2066	–	–	–	–	–	–
	2004	6	1985	53	1918	–	–	–	–	3	190	14	1969	–	–	–	–	–	–
	2005	8	2052	56	1987	–	–	–	–	6	1892	17	2127	–	–	–	–	–	–
	2006	7	2076	57	2141	–	–	0	1469	8	1772	20	2155	–	–	–	–	–	–
	2007	7	2035	55	2105	–	–	0	1543	10	1828	18	2140	–	–	–	–	–	–
	2008	7	1923	61	1763	–	–	0	1436	7	2193	15	2370	–	–	–	–	–	–
	2009	8	4311	62	3824	–	–	0	3459	10	3943	18	4130	–	–	–	–	–	–
	2010	9	4929	62	4429	–	–	0	4025	9	4547	18	4815	–	–	–	–	–	–
	2011	8	5661	63	5074	–	–	0	4640	10	5182	18	5564	–	–	–	–	–	–
	2012	9	6390	63	5846	–	–	0	5182	13	5663	17	6241	–	–	–	–	–	–
	2013	10	7166	63	6648	–	–	0	6251	15	6586	17	6998	–	–	–	–	–	–
	2014	9	7274	63	6637	–	–	0	6367	11	6221	17	6921	–	–	–	–	–	–
	2015	11	6052	66	5117	–	–	0	5497	12	5169	16	5812	–	–	–	–	–	–
	2016	10	23166	63	21614	–	–	0	22762	10	21846	17	22883	–	–	–	–	–	–
	2017	11	30739	63	28647	–	–	0	30074	11	27925	18	30185	–	–	–	–	–	–
United States	1999	–	–	43	7687	12	932	0	6096	2	6779	5	7644	5	3455	–	–	–	–
	2000	–	–	44	9611	16	2074	0	8228	2	8570	7	9595	4	6080	–	–	–	–
	2001	–	–	44	10512	19	2278	0	8515	2	9362	10	10404	4	7594	–	–	–	–
	2002	–	–	44	10904	17	2636	0	9105	3	9553	12	10838	4	7917	–	–	–	–
	2003	–	–	46	11231	18	2652	0	9130	3	9907	14	11205	3	8751	–	–	–	–
	2004	–	–	48	12346	19	2435	0	10205	4	11291	17	12534	3	10395	–	–	–	–
	2005	–	–	49	12077	20	1788	0	10078	4	11372	20	12317	3	10861	–	–	–	–
	2006	–	–	52	10431	24	1767	0	9728	4	10233	23	10739	3	9832	–	–	–	–
	2007	–	–	53	10157	27	1925	0	9390	5	9980	25	10445	3	9630	–	–	–	–
	2008	–	–	54	9224	30	1705	0	8798	6	8973	27	9459	3	9163	–	–	–	–
	2009	–	–	54	8072	26	1264	0	7761	7	8100	28	8427	5	8151	–	–	–	–
	2010	–	–	53	7532	21	1481	0	7631	8	7631	27	7985	6	6996	–	–	–	–
	2011	–	–	55	7587	20	1620	0	6962	10	7170	28	8086	5	5224	–	–	–	–
	2012	8	4400	45	8240	18	801	1	7673	8	7967	22	8503	4	5245	–	–	–	–
	2013	15	9203	–	–	–	–	0	9203	10	9203	31	9203	8	9203	–	–	–	–
	2014	15	11071	–	–	–	–	0	11071	12	11071	31	11071	9	11071	–	–	–	–
	2015	17	13032	–	–	–	–	0	13032	15	13032	33	13032	8	13032	–	–	–	–
	2016	15	16453	–	–	–	–	0	16453	15	16453	31	16453	6	16453	–	–	–	–

**FIGURE 2:**
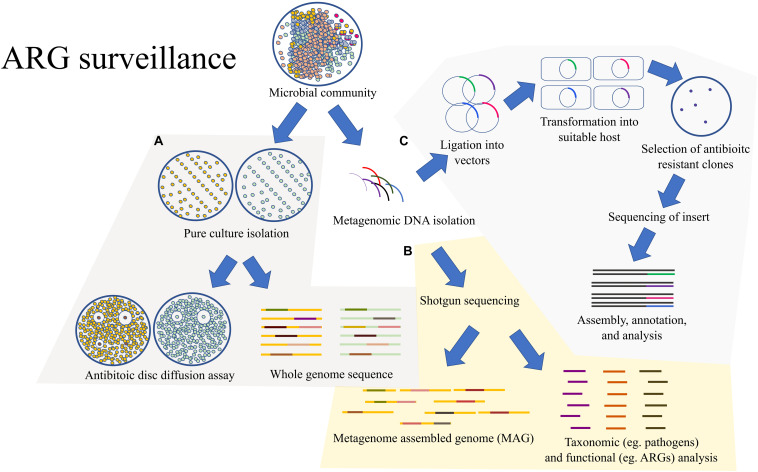
An outline of the methods for surveillance of ARGs: **(A)** A common traditional approach would rely on the pure culture isolation of the MDR bacteria followed by antibiotic susceptibility test through the twofold serial dilution method. The pure cultures could also be sent for WGS. **(B)** Rather than isolating pure cultures, the microbial community could be directly processed for shotgun metagenomic analysis which would provide metagenome assembled genomes (MAGs) and valuable information on the taxonomic composition and functional potential of the overall microbial community. **(C)** The microbial community can also be directly processed for expression-based functional metagenomics for detection of ARGs which might be missed by the shotgun metagenomic approach due to the lack of homology.

In this review, we explore the applications of whole genome and metagenomes in the surveillance of antibiotic resistant genes in detail (Figure 2), followed by the recent updates in combinatorial drug treatments, nano-particles as antibiotic adjuvants, and drug–microbiome interactions.

## Resistome Surveillance Through Whole-Genome Sequencing

Since most pathogens are becoming resistant to a majority of conventional antibiotics, the surveillance of antibiotic resistant genes (ARGs) is an important step in monitoring the spread of antibiotic resistance and its emergence. ARG can be determined by a variety of techniques like microarray, polymerase chain reaction (PCR), and whole-genome sequencing (WGS). Despite advancements in genomic science, detection of antibiotic resistance in clinical laboratories relies on the traditional twofold serial dilution and disk diffusion methods (Hendriksen et al., 2019a). These techniques have been proven to be effective in the formulation of anti-infective therapy. However, for the surveillance of ARGs, such traditional techniques have several disadvantages, such as lack of valid methods for several microbes, differences in laboratory conditions, and also the limitation in the number of tested drugs. ARG surveillance also requires several other pieces of information, such as the comparison of genotypes from different host and environments for route tracking and the degree of dissemination (McDermott et al., 2016; Karp et al., 2017).

Whole-genome sequencing provides the finest level of resolution that comprises all genes within the genome that can be processed for microbial identification, phylogenetic relationships, detecting mutations, putative novel genes identification, retrospective analysis, and predicting phenotypic antibiotic susceptibility. The genome sequence can also provide valuable information on antibiotic resistance traits and its mobility. This is invaluable information in the investigation of outbreaks and real-time surveillance of ARG dissemination (Hendriksen et al., 2019a). Real-time surveillance of ARGs would aid in early recognition of outbreaks and also in the investigation of public health policies (Alghoribi et al., 2018). WGS has helped in several areas, such as detecting the source of outbreaks, track back studies, and forming policies in recalling contaminated food. WGS analysis also provides several other advantages over the traditional resistance testing, such as the predictions of horizontal gene transfer, co-resistance to other antibiotic including heavy meals, and its presence in chromosome or plasmids (Hendriksen et al., 2019a). WGS is currently being used mostly for epidemiological studies since it provides a higher resolution through single-nucleotide variants (SNVs) and multi-locus sequence typing (MLST) than the traditional typing tools (De Been et al., 2015; Schürch and van Schaik, 2017).

The surveillance of ARGs is an important part of the Global Antimicrobial Resistance Surveillance System (GLASS) (World Health Organization, 2015), which aims to generate standardized data that can be compared between nations, track the emergence and spread of ARGs, and serve as guidelines in forming appropriate policies and the allocation of required resources to tackle the ARG threat (Grundmann and Gelband, 2018). In the US, surveillance of ARGs from agriculture, animal, and human health has been taken up by the National Antimicrobial Resistance Monitoring System (NARMS) as a “one health” approach to monitor ARGs and track the transmission route from food that causes human illness (Karp et al., 2017). Similarly, initiatives have already been taken up by European Centre for Disease Prevention and Control (ECDC) in the EU for tracking and investigation of outbreaks (European Centre for Disease Control, 2016). Recent reports from the European Committee on Antimicrobial Susceptibility Testing (EUCAST) have concluded that the WGS-based ARG prediction could soon replace the phenotypic antimicrobial susceptibility testing (AST) for environmental ARG surveillance, which will have no negative impact on individual patients (Ellington et al., 2017). Although several measures have been put forward to address the challenges posed by antibiotic resistance, to overcome the antimicrobial resistance (AMR) crisis, or to at least decelerate the emergence and spread of ARGs, multiple scientific fields, including policy measures and public education, are still important to reduce the inessential usage of antibiotics (Schürch and van Schaik, 2017).

Recent application of WGS for real-time surveillance of multidrug resistant (MDR) pathogens in hospitals have shown that the results are precise with a low turnaround time and are also cost effective (Mellmann et al., 2016). WGS has also been shown to be sensitive in the identification and profiling of antibiotic resistance phenotypes (Bradley et al., 2015; Aanensen et al., 2016). It is also cost effective and provides results quicker than the traditional phenotypic typing, especially for slow-growing pathogens such as *Mycobacterium tuberculosis*. WGS can also detect pathogens in polymicrobial samples with higher sensitivity than the culture technique (Hasman et al., 2014). WGS also enables the identification of not only ARGs but also the types of alleles. Recent WGS studies have shown an 100% correlation between genotype and phenotypic resistance to tetracycline, ciprofloxacin/nalidixic acid, and erythromycin, indicating that the WGS could be a reliable tool for prediction of antibiotic resistance (Zhao et al., 2016). In another study, WGS analysis of 640 *Salmonella* isolates identified over 99% correlation between resistance genotypes and phenotypes with an 100% match for macrolide, quinolones, and tetracyclines, except for aminoglycosides and beta-lactams (McDermott et al., 2016). WGS has also been implemented for comparative analysis from contrasting environments. Carbapenem resistant and ESBL-producing *K. pneumoniae* was found to carry a higher diversity of carbapenemases genes in wastewater isolates while antiseptic-resistance gene *qacE* was exclusive to clinical samples (Surleac et al., 2020).

Dissemination of ARGs from food and animals to humans remains as a major source of illness. WGS of *K. pneumonia* isolates from clinical and retail meat, such as turkey, chicken, and pork in Arizona, United States, exhibited close genetic relatedness, with the latter isolates having a higher likelihood of MDR traits, indicating that there is hardly any resistance in the transmission route from meat to humans (Davis et al., 2015). Similarly, a retrospective analysis of ∼24 000 *E. coli* and *Klebsiella* spp. genomes from clinical and poultry meat in the United Kingdom identified *mcr*-1 gene on plasmids with IncHI2, IncI2, and IncX4 replicon types (Doumith et al., 2016). Furthermore, *mcr*-1 was also detected in *E. coli* ST131 isolates from chicken meat, which is a rare strain among animal *E. coli*, although common in human *E. coli* urinary tract infections (Hasman et al., 2015).

The WGS study on the outbreak of Carbapenem-producing MDR *K. pneumonia* in the ICU of Shanghai Huashan Hospital in east China revealed that the isolates belonged to ST11 CP-Kp. However, single nucleotide polymorphism (SNP) and phylogenetic analysis indicated that they were of different origins, mainly grouped into two different clades of origination from two index patients (Chen et al., 2019a). Similarly, WGS of *E. coli* and *Klebsiella* isolates from patients admitted to the ICU in Maryland Medical Center (UMMC) in Baltimore were identified with bla_*FOX–5*_ gene encoded in IncA/C plasmid. However, they had initially tested negative for AmpC β-lactamase, indicating that the resistance could have been acquired in the ICU (Hazen et al., 2014). The spread of ARGs in an intercontinental scale has also recently been observed through WGS analysis of over 1,832 *Salmonella enterica* serovar Typhi (*S. Typhi*) from 21 countries, particularly in Asia and Africa (Wong et al., 2015). Multiple transfers of a dominant pathogenic lineage, H58, from Asia to Africa in multiple occasions were inferred from the WGS analysis. They also harbored MDR genes integrated into the chromosome as well as in the IncHI1 plasmids (Wong et al., 2015).

## Metagenomic Approaches for Resistome Surveillance

Although whole-genome sequencing is a powerful tool for ARG surveillance, its requirement for pure culture is a major setback, since the majority of the microbes are yet uncultured in the laboratory condition (Steen et al., 2019). This can be sustainably circumvented with the aid of metagenomics. Whole metagenome is the total environmental DNA sequenced through shotgun sequencing technologies bypassing the culturing step. Hence, it offers identification of, theoretically, all viable or non-viable microbes that are culturable as well as those that are not yet cultured It also helps to predict the functional potential and derive the whole-genome sequence. In addition, vast taxonomic data can be analyzed to predict species interactions and the keystone species. The generated data can also be shared globally through various metagenome repositories. In addition, the minimum requirements for infrastructure, laboratory equipment (excluding the NGS sequencer), and the comparatively less controlled environment compared to conventional microbiological laboratories offer a promising future to implement it in low income countries. Hence, metagenomics offers the potential for global surveillance of ARG at a population scale and compared with other environments, which would provide an unprecedented level of information that could serve as guidelines in environmental monitoring and policy making (Duarte et al., 2020). The advancement in shotgun metagenomics has revealed several novel ARGs. Metagenomic data samples from the Red Sea expedition sequenced with GS FLX pyrosequencer identified two novel ARGs, class A beta-lactamase and a thermostable 3’-aminoglycoside phosphotransferase, and its activity were validated *in vitro* (Elbehery et al., 2017).

Shotgun metagenomics offers high throughput analysis of multiple samples. It mainly depends on the homology-based alignment of the sequence with known genes in a database. Hence, in the last few years, several tools and pipelines have been developed (Table 2) (Niu et al., 2017). A typical reference-based workflow would involve quality assessment of the raw NGS data that includes adaptor removal, trimming of reads with a low phred score, and screening out reads below the minimum threshold length, and could also be followed by removal of the host genome or other unwanted contaminants and a dereplication step. Several tools are available to aid in the quality assessment, such as Cutadapt (Martin, 2011), FastQC (Andrews, 2010), Trimmomatic (Bolger et al., 2014), and fastp (Chen et al., 2018). The clean reads can be directly mapped against a reference database for annotation using bowtie2 (Langmead and Salzberg, 2013), BWA (Burrows-Wheeler transform) (Li and Durbin, 2009), or KMA (k-mer alignment) (Clausen et al., 2018). Direct mapping of the reads could lead to higher false positives. However, they are computationally less demanding and more sensitive to low abundant genes (Boolchandani et al., 2019). Although direct mapping of the clean reads have advantages, a higher accuracy in ARG detection can be achieved through an assembly based method, which also offers information on the genes flanking to the gene of interest (Lal Gupta et al., 2020). In *de novo* assembly, the quality controlled reads are assembled into contigs using assembler programs such as MEGAHIT (Li et al., 2015), SPAdes (Bankevich et al., 2012), Velvet (Zerbino and Birney, 2008), metaSPAdes (Nurk et al., 2017), IDBA-UD (Peng et al., 2012), and Meta Ray (Boisvert et al., 2012). The contigs are further processed for open reading frame (ORF) prediction using FragGeneScan (Rho et al., 2010) or OrfM (Woodcroft et al., 2016). The predicted ORFs are then aligned to the appropriate ARG database using DIAMOND (Buchfink et al., 2015), VSEARCH (Rognes et al., 2016), or USEARCH (Edgar, 2010). The *de novo* assembly based approach, despite its several advantages, could lead to data loss on rare genes. To overcome the limitations of read and assembly based methods, long sequencing technologies, such as Pacific Biosciences (PacBio) and Oxford Nanopore, were developed. The read length in NGS has increased substantially in recent years. Assembly of reads, from long reads NGS technologies to short reads, provides a longer continuous or semi-continuous genomic fragment (contigs or scaffolds) that has several advantages. It allows identification of full-length gene(s), gene clusters, polyketide synthase (PKS), and also identification of taxa at a higher resolution than that on unassembled reads (Vollmers et al., 2017). Although assembly provides such benefits, it requires fairly deep sequencing. Metagenomes are also composed of uneven microbial compositions that lead to uneven sequencing depths of the species/strains. This is particularly difficult for the rare species. Although pure cultures can be grown clonally, microbial communities in natural environments often harbor non-clonal cells, which makes it difficult to impose strict parameters in the assembly of overlapping reads (Breitwieser et al., 2018).

**TABLE 2:** Bioinformatics tools for ARG annotation from whole genome and metagenome datasets.

S. No	Bioinformatic tool	Web address	Description	Updated date	References
1	ResFinder	https://cge.cbs.dtu.dk//services/ResFinder	Identifies acquired and chromosomal mutation from shotgun reads	02-06-2020	Zankari et al., 2012
2	ARGs_OAP	https://smile.hku.hk/SARGs	Identifies ARGs in metagenomic data thought galaxy server	2020.07.15	Yin et al., 2018
3	ARDB	http://ardb.cbcb.umd.edu/	A database of ARGs. However, the ARDB is no longer maintained	July 3, 2009.	Liu and Pop, 2009
4.	The Comprehensive Antibiotic Resistance Database (CARD)	https://card.mcmaster.ca/	A database of ARGs that are peer reviewed. It includes software to predict resistome from protein, genome, or metagenomics datasets	Monthly updated	Alcock et al., 2020
5	ARGO - antibiotic resistance genes online	http://bioinformatics.org/argo/beta/submissions/submit_sequence.php	A database of ARGs with majority of sequence from β - lactamase	NA	Scaria et al., 2005
6.	DeepARG	http://bench.cs.vt.edu/deeparg	It provides web service and command line tools for prediction of ARGs from metagenome data using machine learning approach	NA	Arango-Argoty et al., 2018
7.	Graphing Resistance Out Of meTagenomes (GROOT)	https://github.com/will-rowe/groot	It provides a resistome profile from metagenomic datasets	May 11 2020	Rowe and Winn, 2018
8.	KmerResistance	https://cge.cbs.dtu.dk/services/KmerResistance/	It provides resistome profiles from whole genome sequence using k-mer approach	March 17, 2020	Clausen et al., 2018
9	AMRFinderPlus	https://www.ncbi.nlm.nih.gov/pathogens/antimicrobial-resistance/AMRFinder/	It focused on acquired or intrinsic AMR genes through HMM approach. It also detects other class of resistance genes such as heavy metal resistance genes.	May 13 2020	Feldgarden et al., 2019
10	RAST and PATRIC	https://rast.nmpdr.org/ https://patricbrc.org/	It provides genotypic and phenotype prediction in genomes including the identification of genomic regions relating to ARGs	NA	Davis et al., 2016
11	ARG-ANNOT	http://www.mediterranee-infection.com/article.php?laref=282&titer=arg-annot	It detects existing and putative new ARGs in bacterial genomes	Monthly	Gupta et al., 2014
12	sraX	https://github.com/lgpdevtools/srax	A fully automated tool for resistome analysis in bacterial genomes. The results are displayed in HTML formatted files.	Feb 5 2020	Panunzi, 2020
13	Abricate	https://github.com/tseemann/abricate	In scans contigs for antimicrobial resistance or virulence genes. However, it does not resistance due to point detect mutations.	Mar 28 2020	Zong et al., 2018
14	Search Engine for Antimicrobial Resistance	http://computing.bio.cam.ac.uk/sear/SEAR_WEB_PAGE/SEAR.html	A web based tool for detection of horizontally acquired ARGs from raw metagenomic reads	NA	Rowe et al., 2015
15	Sequence-Search-Tool-for-Antimicrobial-Resistance-(SSTAR)	https://github.com/tomdeman-bio/Sequence-Search-Tool-for-Antimicrobial-Resistance-SSTAR-	A standalone software written in java (hence platform independent) that predicts ARGs from WGS. It also notifies users if it detects a truncate a possible ARG.	Sep 11, 2019	de Man and Limbago, 2016
16	MEGARes	https://github.com/cdeanj/resistomeanalyzer	A database that includes ARGs as well as biocide and heavy metal resistance genes	Aug 1, 2018	Lakin et al., 2017
17	Multiple Antibiotic Resistance Annotator (MARA)	http://mara.spokade.com	Multiple Antibiotic Resistance Annotator (MARA) database is mainly focused on the gram negative mobile ARGs	NA	Partridge and Tsafnat, 2018
18	FARME DB	http://staff.washington.edu/jwallace/farme	Functional Antibiotic Resistant Metagenomic Element (FARME) database focus on the mobile elements are the flanking genes	NA	Wallace et al., 2017
19	ARGA	http://mem.rcees.ac.cn:8083/	It consists of a database of antibiotic resistance gene with the main focus on ARG primer designing	Mar 25th, 2019	Wei et al., 2019
20	ARGminer	http://bench.cs.vt.edu/argminer	An online platform that enables curation of ARGs through crowdsourcing	NA	Arango-Argoty et al., 2020
21	ResistoMap	http://resistomap.rcpcm.org/	An interactive web based tool for visualization of ARGs in human gut micorbiome	NA	Yarygin et al., 2017
22	ARGs-OSP	http://args-osp.herokuapp.com	ARGs-OSP (antibiotic resistant genes-online searching platform) is an online ARG search platform with over 50 thousand WGS and more than 800 metagenoms	NA	Zhang et al., 2020
23	LRE-Finder	https://cge.cbs.dtu.dk/services/LRE-finder/	A web tool to study the mutation in 23S rRNA gene and linezolid resistance using whole-genome sequence data.	NA	Hasman et al., 2019
24	MvirDB	http://mvirdb.llnl.gov/	It is a database of antibiotic resistance gene along with protein toxins and virulence factor	NA	Zhou et al., 2007
25	MUBII-TB-DB	http://umr5558-bibiserv.univ-lyon1.fr/mubii/mubii-select.cgi	A database of mutations that confer resistance to ARGs in *Mycobacterium tuberculosis*	NA	Flandrois et al., 2014

Metagenomics have already been implemented in the surveillance of ARGs in various environments. Metagenomic studies of built environments have shown that dust in homes harbors significantly higher ARGs compared to drinking water (Maamar et al., 2020). The authors also detected plasmid borne ARG *lnuA* (resistance to lincosamides) which was further confirmed *in vitro*. Build environments, such as biogas plants, posed a significant threat since the digested residue is used as biofertilizers in agriculture and commercial manures. Metagenomics surveillance of ARGs revealed that the thermophilic biogas reactors have a higher ARG removal efficiency than the mesophilic biogas (Luo et al., 2017). Metagenomics studies have also shown ARG enrichment in animal farming upon antibiotic administration (Munk et al., 2017). Nonetheless, ARGs in the gut microbiome of farm animals are well known reservoirs of ARGs, such as *tetW*, *tetQ*, *mefA*, *ermB*, qepA, *qnrB*, and, *cfxA2*, despite the lack of antibiotic selective pressure (Joyce et al., 2019). In addition to animal farming, aquaculture, such as mariculture, has gained considerable interest in recent years. A metagenomic study of mariculture in China identified a similar co-occurrence pattern of ARGs in Proteobacteria and Bacteroidetes. They also identified that *Nitrospinae* (nitrifying bacteria) were prime ARG hosts (Wang et al., 2018).

Among all reservoirs of ARGs, wastewater treatment plants (WWTPs) remain one of the major conduits of ARGs. Recent studies in Indian WWTPs from small-scale industries were found to be dominated by “ESKAPE pathogens” of MDR genotype; IncP-1 plasmid pKJK5 and pB8 were also detected (Yadav and Kapley, 2019). Similarly, sewage in Montevideo had significantly higher diversity of ARGs, such as TEM-4 and TEM-33 betalactamases, than the beach samples (Fresia et al., 2019). ARGs’ abundance were also found to increase in winter (1.79 × 10^12^ copies/L) rather than during summer (3.27 × 10^11^ copies/L) in Chinese WWTPs but no differences were observed based on geography (Su et al., 2017). ARG diversity could also be determined by the types of treatments in the plant. For instance, activated sludge harbor lower (*n* = 42) ARGs than digested sludge (*n* = 51) (Guo et al., 2017). Nonetheless, both sludge types harbor plasmids and other mobile elements such as transposons, integrons (intI1), and insertion sequences. Environmental microbes, such as *Clostridium* and *Nitrosomonas*, were also found to be the host of multiple ARGs (Guo et al., 2017). Although WWTP influent have high diversity and abundance of ARGs, it is expected since they represent waste disposal of a large population that would include human and animal excreta. The core resistome of the WWTPs were associated to the core microbial community of the WWTP, as well as the human gut microbiome, indicating the influence of humans on the ARGs in the WWTP (Ng et al., 2017; Su et al., 2017). In contrast, the effluent of WWTPs are treated for the removal of pathogens and other microbes. Hence, ARGs should reduce drastically. In line with this, ARGs in the wastewater of hospitals and municipals had reads as high as 197,816 x/Gb which reduced significantly to 2,692 x/Gb after wastewater treatment, which is lower than that of surrounding natural water bodies (7,985 x/Gb) (Ng et al., 2017). However, the core ARGs in the metagenome (wastewater and surface water) were composed of multidrug resistant efflux pumps, resistance to aminoglycoside, macrolide-lincosamide-streptogramins (MLS), quinolones, sulfonamide, and tetracycline resistance. ARGs such as bla_*KPC*_, bla_*CTX–M*_, bla_*SHV*_, and bla_*TEM*_ were significantly over represented in the wastewater, which were encoded in Tn3-based transposon (Tn4401), indicating their mobility (Ng et al., 2017). Research on virome in hospital wastewater has shown an interesting finding that the phage DNA (0.26%) harbored a higher relative abundance of ARGs than the bacterial DNA (0.18%), particularly the ATP-binding cassette (ABC), Resistance-Nodulation-Cell Division (RND), β-lactamases, plasmid-mediated quinolone resistance, and beta lactam resistance genes (Subirats et al., 2016).

Antibiotic resistant gene surveillance has been aided by tools such as SRST2 (Inouye et al., 2014) and KmerResistance (Clausen et al., 2016), which are widely used for the prediction of ARGs that use alignment and the k-mer approach. However, the setback of such tools is their inability to detect mutations and SNPs that render resistance to antibiotics. Mutations in antibiotic target genes pose a significant threat. Such mutations can be identified from genomes and metagenomes with tools such as Mykrobe predictor (Bradley et al., 2015) and PointFnder (Zankari et al., 2017). Mykrobe predictor utilizes the k-mers approach to identify ARG variants in *S. aureus* and *M. tuberculosis*. The PointFinder has two databases that stores information regarding the position(s) of mutated codon(s) and the chromosomal gene database. Using BLASTn, query sequences of ≥80% identity are processed for analysis against the chromosomal mutation database. Recent developments in ARG variant detection includes tools such as Mumame (Mutation Mapping in Metagenomes) (Magesh et al., 2019) and ARIBA (Antimicrobial Resistance Identification By Assembly) (Hunt et al., 2017). ARIBA is a command line tool that accepts paired end reads and detects acquired ARGs, mutations such as deletions, and SNPs (synonymous and non-synonymous).

In addition to the alignment approach, hidden Markov models (HMMs) have been implemented in resistome studies in metagenomic data. The HMM approach involves the initial translation of nucleotide sequences into all six reading frames. The translated sequences are then input for HMM analysis using HMM models constructed with experimentally verified ARG sequences. Using this approach, 12.7 terabytes of genomic data, including metagenomes and nucleotide sequences from NCBI nucleotide database and NCBI RefSeq bacterial genomes, were screened and 362,843 sequences were identified as *qnr* genes (quinolone resistance genes) (Boulund et al., 2017). Among them, 611 sequences were above 200 bp and 52 were novel *qnr* genes. The results were reinforced by experimental validation of 20 *qnr* genes from the study through gene synthesis and expression in *E. coli*. In another metagenomic study from hospital effluent in Mumbai, India, 112 ARGs and several mobile genetic elements were identified. HMM-based analysis further identified novel metallo-beta-lactamase (MBL) genes under subclass B1 (*n* = 10), B2 (*n* = 1), and B3 (*n* = 10), which were further verified experimentally (*n* = 6) (Marathe et al., 2019). It is also important to note that the general definition of novel ARG considers a gene to be novel if it has ≤79% amino acid sequence similarly to known ARGs (Levy et al., 1999; Chopra and Roberts, 2001). Hence, several studies have considered ARGs as novel when the amino acid sequence similarity is below 80% (Berman and Riley, 2013; Berglund et al., 2019). However, amino acid sequence identities above 80% have also been considered as novel ARG (i.e., *mcr*-10 and aar-2) (Yong et al., 2009; Wang et al., 2020).

Homology alignment methods rely on the “best hits” against known ARGs in an existing database, which leads to a high rate of false negatives due to the high cut-off. To overcome such a limitation, machine learning approaches have provided an alternative strategy to the prediction of ARGs. Machine learning in antibiotic resistance prediction requires datasets of known ARG sequences from which the algorithm determines the common characteristics, known as “features,” from the input ARGs (Chowdhury et al., 2020). Machine learning approaches have been considerably successful in ARG prediction and were able to predict ARGs which “best hits” homology alignment failed in identifying (Arango-Argoty et al., 2018). However, machine learning is a considerably new addition in the toolbox of ARG prediction strategies, which also requires a large set of known ARGs to train the models. Hence, the robustness of machine learning approaches are dependent on the underlying model as well as the amount of ARGs in the training dataset. One of the most common machine learning models for ARG prediction from metagenomes is DeepArg (Arango-Argoty et al., 2018). It was trained against ARGs from the Antibiotic Resistance Genes Database (ARDB) (Liu and Pop, 2009), the Comprehensive Antibiotic Resistance Database (CARD) (Jia et al., 2017), and manually curated ARGs from the Universal Protein Resource (UNIPROT) database (Apweiler et al., 2004). Two models were developed: DeepARG-SS for short reads and DeepARG-LS for long sequences. The DeepARG-SS was constructed to predict ARGs from short read sequences while DeepARG-LS predict ARGs from full gene length sequences. The models are trained for over 30 ARG classes with high accuracy. The authors tested the ability of the model against 76 experimentally validated novel metallo beta lactamase genes (Berglund et al., 2017) with 85% accuracy, indicating that the model is capable of predicting novel ARGs. Furthermore, the traditional best hit approach would fail to predict the novel metallo beta lactamase genes, indicating the sensitivity and robustness of machine learning approaches in ARG surveillance (Arango-Argoty et al., 2018). DeepArg has been widely used in several studies that have revealed correlations of total ARGs with anthropogenic impacts in natural environments (Collins-Fairclough et al., 2018; Chen et al., 2019b; Zeng et al., 2019), increased ARGs in murin gut under antibiotic treatments (Korry et al., 2020), and reduction of ARGs in bioreactors upon ozone pre-treatment (Xia et al., 2020).

Short reads from Illumina sequencing remains one of the most used sequencing technologies for metagenomic studies. A novel tool, fARGene, was recently introduced that could identify ARGs from short reads without the need for assembly (Berglund et al., 2019). The tool relies on the optimized gene models that enable the detection of low similarity ARGs or those which were previously uncharacterized. The authors identified 221 β-lactamases from five metagenomic datasets, out of which 81% of 38 novel ARGs, reconstructed through fARGene, were experimentally verified to confer resistance in *Escherichia coli*. fARGene have been implemented in resistome studies on migratory birds that revealed several novel β-lactamase genes (Cao et al., 2020).

Despite the robustness of such tools, their reliance on sequencing alone poses a major weakness in characterizing ARGs of distant homologies in metagenomic datasets. To overcome this, a pairwise comparative modeling (PCM) system was developed based on 3D structural alignment against known antimicrobial resistance proteins. This enabled the prediction of ARGs of distant homologs to known proteins (Ruppé et al., 2019). This tool is based on the fact that active sites between similar proteins would be conserved, which might not be detected by protein sequencing alone. Using this tool, analysis of over 3 million proteins from human fecal samples predicted 6,095 ARDs (antibiotic resistance determinants) while BLASTP(Altschul, 1990) and DeepARG predicted 67 and 2,139 ARDs respectively, indicating its superiority. Interestingly, the authors also observed that the abundance of some pdARD (predicted ARDs) families declined upon combination of antibiotics treatments. However, the model suffers for predicting ARGs such as mutations in housekeeping genes, and efflux pumps (*TetA*, *QepA*, and, *mcr*-1).

The surveillance of AMR through the metagenomic approach required a harmonized method that should be available for coordinated collaboration between surveillance programs. Massive Open Online Course (MOOC) on ARG detection through metagenomics are available to impart knowledge to the researchers that would serve as a reference material (Duarte et al., 2020).

## Discoveries in Novel Antibiotics Resistant Genes Through Expression Based Metagenomic

Functional metagenomics was initially proposed as a novel method to explore the vast uncultured microbial community (Handelsman et al., 1998). Functional metagenomics has emerged as a promising tool for biomining of novel genes, peptides, and secondary metabolites (Johnson et al., 2017). Functional metagenomic pipelines consist of environmental DNA (eDNA) isolation followed by shearing into the desired size. For a small insert library, eDNA is sheared up to 10 kb, while for a large insert library, the insert can range up to 50 kb (Nora et al., 2019). Sheared eDNA is generally blunted and ligated on to a suitable vector and transformed into a suitable host for heterologous expression. Clones with the desired phenotype are selected in a selective media. Functional metagenomics have several advantages over other techniques, such as shotgun metagenomics. It enables the screening of desired genes from the total microbial community, which is not possible with culturable techniques since the majority of the microbes are as yet uncultured. Furthermore, the high throughput nature of the functional metagenomics ensure maximum sequences are screened. The library can be stored, replicated, and reanalyzed for reproducibility. It is also applicable to every environmental sample. Functional metagenomics outperforms shotgun metagenomics since the latter relies on alignment with known genes within a database while the former is independent of any prior knowledge on the sequence information.

With the implementation of functional metagenomics, several novel ARGs have been reported from a diverse set of host and environmental samples (Forsberg et al., 2012; Pehrsson et al., 2013; Ngara and Zhang, 2018). Recently, functional metagenomics from environmental samples identified novel β-lactamase genes BlaCX1 (B1 subclass), BlaCM1 (class A), BlaH33 (B3 subclass), blaRM3 (B3 subclass), and blaAM1 (B3 subclass) (Zhang et al., 2019). Furthermore, a transposase gene, ISPme1, was also identified upstream of BlaCM1, indicating that the antibiotic resistant gene might be mobile. Novel sulfonamide resistance genes AEW9_dhps01, SEW2_dhps01, and SEW5_dhps01, and AEG2_DHPS01 were also detected in forest and grassland soils from Germany, which were relatively unexposed to human intervention (Willms et al., 2019). The study also identified major facilitator superfamily (MFS) efflux pump mediated tetracycline resistance. Multidrug efflux pumps represent a major mechanism in antibiotics resistance in several ecosystems (Imchen et al., 2018). Its broad array of binding ions and compounds for detoxification remains a major obstacle in the fight against antibiotic resistance. Furthermore, recent expression-based metagenomics have revealed 34 novel ARGs in Canadian agricultural soil, most of them representing (multi)drug efflux systems, including PPPAZI-4, that putatively encodes a novel macrolide resistance mechanism (Lau et al., 2017).

Although functional metagenomics is one of most robust approaches for novel gene discoveries without any prior information of the sequence, it has its own limitations. The expression of the insert is dependent on its functionality within the host. For the expression of the desired phenotype, full clusters of the required gene have to be captured in the insert and expressed. Furthermore, expression of foreign gene(s) can also cause perturbation of the host transcriptional profile (Warren et al., 2008), toxicity to the host (Wang et al., 2019), and incompatibility for expression (Jung et al., 2012) due to several factors (such as biased G/C contents) (Pehrsson et al., 2013). For instance, expression of novel antibiotic-resistant genes in *E. coli* BL21 (DE3) was reported to fail in the sub-cloning due to the toxicity caused by over production of the enzymes while *E. coli* EC100 successfully expressed the ARG (Zhang et al., 2019). To circumvent these drawbacks, several ranges of hosts and vectors may be used such as *Bacillus subtilis* (Westers, 2004), *Streptomyces lividans* (McMahon et al., 2012), *Pseudomonas putida* (Nagayama et al., 2015), *Burkholderia graminis*, *Agrobacterium tumefaciens*, *Caulobacter vibrioides*, and *Ralstonia metallidurans* (Craig and Brady, 2011). Although usage of multiple hosts would mean a substantial increase in workload, it does provide higher hits.

In order to circumvent the aforementioned limitation, a metagenomics-guided strategy was introduced recently that bypasses the library preparation step. It involves the regular sequence-based metagenomic detection of ARGs followed by PCR amplification of ARGs with moderate homology (∼40%). The PCR amplicons are cloned directly into a suitable host to validate its expression (Yang et al., 2017). Using this approach, 76 ARGs were identified, out of which 25 novel ARGs with 40-70% homology were validated *in vitro*.

Another approach recently introduced is the metagenomic cloning of rRNA genes in *E. coli* Δ7. This particular strain of *E. coli* has a functional compatibility with rRNA genes from distant phylogenies. Hence, introduction of metagenomic rRNA into the *E. coli* would render it resistant to spectinomycin if the insert has resistance-conferring mutations. Using this technique, three novel resistance genes, U1183C, C1063U, and U1189C, were identified. Interestingly, introduction of a resistant mutation *in E. coli* 16S rRNA gene rendered the host resistant to spectinomycin, indicating the host independent resistance of the mutation (Miyazaki and Kitahara, 2018). The authors further describe its potential applications in exploration of aminoglycoside resistant mutation, detection of novel resistance mutation from pathogens, and enrichment of samples in antibiotics to study the presence of novel resistance mutations.

## Combinatorial Treatments to Combat Multidrug Resistance

Resistance to antibiotics have increased the dosage recommendation of drugs. However, such an increase in dosage could lead to adverse side effects. A combination of two or more antibiotics with multiple modes of action increases the efficiency and thus reduces the dosage requirement (Table 3). A combination of antibiotics also has synergistic effects and delays the emergence of a drug-resistant mechanism (Tamma et al., 2012). *M. tuberculosis* is a major causative agent of tuberculosis, which is the leading disease in immunosuppressive patients, such as those with acquired immunodeficiency syndrome (AIDS). The combination of isoniazid and rifapentine for a period of 12 weeks was found effective against *M. tuberculosis* (Centers for Disease Control and Prevention [Cdc]., 2011). Similarly, in a small scale study from the University of Pittsburgh Medical Center, out of 21 patients, 15 patients successfully recovered from Multidrug-resistant *Pseudomonas aeruginosa* infections when treated with Ceftolozane-Tazobactam combination (Haidar et al., 2017). However, resistance to Ceftolozane-Tazobactam was developed in three patients within 8 days of treatment, indicating that the resistance to combinatorial drug treatments could be rapid.

**TABLE 3:** Recent formulations of antibiotic combinations with natural compounds towards bacterial pathogen.

*Antibiotic*	*Combination*	*Target to organism*	*References*
*Ciprofloxacin*	Chitosan	*Escherichia coli*	Erman et al., 2017
*Penicillin*	Nisin I4V	*S. pseudintermedius DSM 21284*	Field et al., 2016
*Chloramphenicol*	Nisin, nisin V, and nisin I4V derivatives	*S. aureus SA113*	Field et al., 2016
*Vancomycin*	Propolis extract	*MRSA 10442*	Al-Ani et al., 2018
*Oxacillin*	Propolis extract	*MRSA 10443*	Al-Ani et al., 2018
*Vancomycin*	Propolis extract	*E. faecalis 51299*	Al-Ani et al., 2018
*Levofloxacin*	Propolis extract	*S. pneumoniae 49619*	Al-Ani et al., 2018
*Levofloxacin*	Propolis extract	*H. influenza 49747*	Al-Ani et al., 2018
*Vancomycin*	Propolis extract	*S. pyogenes12344*	Al-Ani et al., 2018
*Ofloxacin*	Calixarene 2	*S. epidermidis ATCC 35984, P. aeruginosa isolate*	Consoli et al., 2018
*Chloramphenicol*	Calixarene 2	*MRSA 15*	Consoli et al., 2018
*Chloramphenicol*	Calixarene 2	*P. aeruginosa ATCC 9027*	Consoli et al., 2018
*Cefuroxime*	Rosehip, pomegranate blossom, rosehip bag	*E. coli*	Hacioglu et al., 2017
*Ampicillin-Sulbactam*	Pomegranate blossom, rosehip bag, rosehip	*E. coli*	Hacioglu et al., 2017
*Ciprofloxacin*	Pomegranate blossom, rosehip, rosehip bag	*E. coli*	Hacioglu et al., 2017
*Ciprofloxacin / Ceftazidime/Amikacin*	Rosehip, pomegranate blossom, green tea, rosehip bag, black tea bag, black tea	*P. aeruginosa*	Hacioglu et al., 2017
*Erythromycin/Ciprofloxacin/Ampicillin*	Rosehip, pomegranate blossom, black tea, green tea, rosehip bag, green tea bag, Thyme, wormwood, mint, sage bag, mint bag, echinacea bag, black tea bag, orengo	*MSSA*	Hacioglu et al., 2017
*Vancomycin/Gentamicin/Ciprofloxacin/Linezolid/Daptomycin*,	P128 (antistaphylococcal protein)	*Staphylococcus aureus*	Nair et al., 2016
*Imipenem*	Colistin	*E. coli, K. pneumoniae*	Dosler et al., 2016
*Cefotaxime/Amikacin*	Bile acid oligomers	*Staphylococcus aureus*	Singla et al., 2018
*Ampicillin/Meropenem/Cefazolin/Cefotaxime/Cefpirome/Cefuroxime*	Pongamia pinnata seed coat extract	*Methicillin-resistant Staphylococcus aureus*	Su et al., 2020
*Cefoxitin/Mupirocin/Co-Trimoxazole/Ciprofloxacin*	Fennel essential oil	*Staphylococcus aureus*	Kwiatkowski et al., 2017
*Oxacillin, Ceftazidime/Fluconazole.*	Alpinia purpurata	*S. aureus and C. albicans*	Ferreira et al., 2018
*Penicillin, Ampicillin/Erythromycin*	Trp-containing antimicrobial peptides (amps)	*Staphylococcus epidermidis*	Shang et al., 2019
*Clarithromycin/Metronidazole*	Hibiscus sabdariffa	*Helicobacter pylori*	Hassan et al., 2016

The widespread prevalence of antibiotic resistance has highlighted the importance of combinatorial therapy using compounds of various origins as adjuvants (Table 2). Secondary metabolites of plant origin, which are non-antimicrobial, have been found to significantly enhance the effectiveness of traditional antibiotics when administered in combination. The combinatorial administration of phytochemical morin, pyrrolidine, and quercetin with ciprofloxacin, tetracycline, and erythromycin against *S. aureus* increases the effectiveness of the antibiotics and reduced biofilm formation (Abreu et al., 2016). This was especially true for *S. aureus* SA1199B overexpressing *NorA* gene. Interestingly, reserpine and quercetin inhibited efflux pump and the application of quinine and morin reversed the antibiotic tolerance induced by sub-lethal concentrations of ciprofloxacin. Phytosterol stigmasterol in combination with ampicillin showed up to 98.7% inhibition of clinical gram positive and gram negative bacteria (Yenn et al., 2017). In addition to phytochemicals, biosurfactants of various origins have also exhibited effective antibiotic adjuvant properties. The amphiphilic nature of the biosurfactants form stable supramolecular complexes with the bacterial phospholipid membrane to creates pores and disturbs the membrane processes and causes cell lysis. Biosurfactants such as rhamnolipids have been found to have synergistic effects with multiple antibiotics against bacteria and fungus (Chen et al., 2017). Adherent biofilm cells formed by *E. coli* CFT073 were successfully removed by treatment with biosurfactant from *Bacillus licheniformis* V9T14 in combination with antibiotics. The combination was more than 90% effective compared to antibiotics or the use of a surfactant alone (Rivardo et al., 2011). Furthermore, dilipid ultrashort cationic lipopeptides (dUSCLs) also enhances the efficacy of several conventional antibiotics, such as chloramphenicol, rifampicin, fosfomycin, ciprofloxacin, and vancomycin, against clinical isolates of MDR *P. aeruginosa*, Enterobacteriaceae, and *Acinetobacter baumannii* (Domalaon et al., 2019). The dUSCLs were found to decrease the MIC (minimum inhibitory concentration) of chloramphenicol 32-fold as a result of membrane permeabilization and active efflux distribution.

Biofilm formation by *P. aeruginosa* is a major pathoadaptation that resists the effectiveness of antibiotic tobramycin therapy in cystic fibrosis (CF) patients. The Food and Drug Administration (FDA) approved triclosan, which was identified as a suitable adjuvant for tobramycin after screening several drug repurposing libraries (*n* = 6080 compounds) (Maiden et al., 2018). The combination reduced MIC of tobramycin 100-fold, and could even kill persister cells and were effective against *Burkholderia cenocepacia* and *S. aureus*. Moreover, triclosan was also found to enhance gentamicin and streptomycin. Non-steroidal anti-inflammatory drugs (NSAIDs) such as aspirin, ibuprofen, and diclofenac have also shown to be effective antimicrobial compounds. Combinations with cefuroxime and chloramphenicol increased the antibiotic activity against MRSA and caused a several-fold reduction in MIC for cefuroxime (up to 8192-fold) and chloramphenicol (up to 64-fold) (Chan et al., 2017). Several other compounds, such as Nebramine-cyclam and EDTA, have also shown synergistic effects with antibiotics against *P. aeruginosa* (Ammeter et al., 2019; Maisetta et al., 2020).

## Nanoparticles as Adjuvants With Antibiotics

**FIGURE 3:**
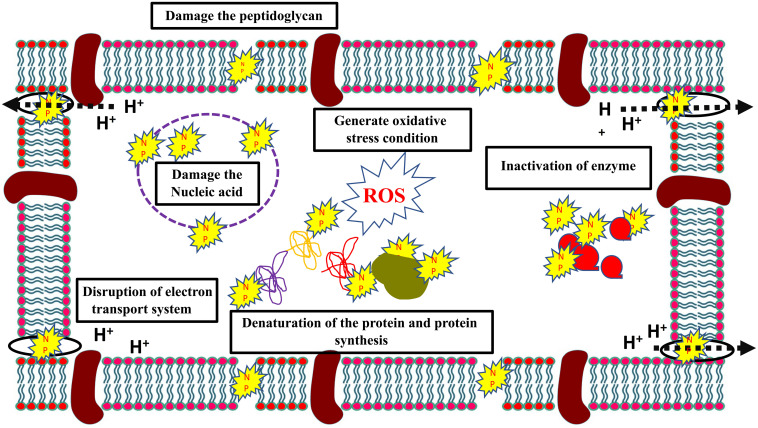
Multiple bacterial inhibitory mechanisms of nanoparticles.

To combat this increased resistance to antibiotics, combination therapy of traditional antibiotics with nanoparticles have been proposed that could increase its effectiveness. Nanoparticles are metallic particles, sized between 1 and 100 nm, with a high surface structure area and high binding properties to the target. Nanoparticles alone or in combination with other antibiotics have shown high antibiotic efficiency against drug resistant bacteria with various modes of action (Figure 3). Various metallic nanoparticles are currently available, such as gold, silver, and copper. Single elemental nanoparticles (SENPs) vary in their antimicrobial activity based on the elemental nanoparticles. For instance, tungsten carbide nanoparticles have a weaker antibiotic activity than silver nanoparticles against hospital acquired *S. aureus* and *P. aeruginosa* (Bankier et al., 2019). However, SENP combinations of tungsten carbide, silver, and copper have shown significant increases in efficiency (Bankier et al., 2019). Silver nanoparticles (AgNPs) in particular are of special interest. Silver nanoparticles combined with polymixin B and rifampicin have a synergistic effect against carbapenem-resistant *Acinetobacter baumannii* ABA1604 *in vitro* as well as *in vivo Acinetobacter baumannii*-infected mouse models (Wan et al., 2016). The MIC of AgNPs (10–12 μg mL^–1^) against several gram +ve and –ve bacteria was also observed to exhibit a synergistic effect in combination with kanamycin by increasing bacterial membrane permeability (Vazquez-Muñoz et al., 2019). The authors observed that the synergistic effect of the combination could be due to the disruption of the cell wall that promotes the antibiotic activity. However, AgNPs and β-lactam antibiotics combinations had no synergistic actions, since β-lactam antibiotics and AgNPs act on the cell walls. Similarly, AgNPs combined with β-lactam (ampicillin and penicillin) did not show a synergistic effect against *Salmonella typhimurium* DT 104 (Deng et al., 2016). On the other hand, AgNPs combined with either enoxacin, kanamycin, neomycin, or tetracycline showed a synergetic effect against *Salmonella typhimurium* DT 104. The authors found that the antibiotics (except ampicillin and penicillin) could form complexes with AgNPs that promoted AgNPs binding to *Salmonella* and promotes Ag+ release, which increased Ag+ concentration around *Salmonella* and inhibited its growth. Hydrophobic AuNPs nanoparticle have also shown synergistic actions with fluoroquinolone against *S. aureus*, *E. coli*, and *P. Aeruginosa* that reduced the MIC of fluoroquinolone 16-fold (Gupta et al., 2017). The synergy was achieved by the downregulation of Tolc-AcrAB efflux pump proteins (*bamA*, *bamD*, and *bamE*), which are involved in detoxification, and also the downregulation of several other proteins involved in lipopolysaccharides synthesis (*rfaQ*) and other important cellular functions.

In addition to the *in vitro* antimicrobial properties of nanoparticles, *in vivo* studies on manganese nanoparticles (MnNPs) in rats (*n* = 60) have also shown antibacterial properties and significant reductions of wound areas in a dose-dependent manner with no non-cytotoxicity (Mahdavi et al., 2020). Copper sulfide nanoparticles (CuS NPs) were also effective against *Aeromonas hydrophila*, *B. subtilis*, and *S. aureus* infection in zebrafish when administered via injection as well as via a medicated bath (Ayaz Ahmed and Anbazhagan, 2017). Furthermore, inspection of the CuS NPs administered to the zebrafish’s liver and brain showed no toxicity and also the CuS NPs exhibited hemocompatibility to human red blood cells. Nanoparticles for human usage, such as Agicoat and Acticoat for dressing of wounds, are currently commercially available. However, a recent study found that such dressing could cause toxicity in the liver but not in the brain, heart, or lungs in rats (Karimi et al., 2020). Silver nanoparticles have also been implemented to combat against periprosthetic joint infection (PJI) with good biocompatibility (Wei et al., 2017). Although there are broad applications of nanoparticles, it should be noted that nanoparticles are known to attack both healthy as well as microbial cells. However, the advantages of nanoparticles could outweigh the toxicity of the nanoparticles. Normal eukaryotic cells are more resistant to the nanoparticles, owing to the immune system and less affinity compared to microbial cells (You et al., 2012).

## Drug–Microbiome Interactions

The influence of antibiotic therapy on the human gut microbiome has been a major area of focus in the past decade. Understanding the impact of antibiotics on the gut microbiome is crucial in order to tackle the ARGs crises. Firstly, similar to increased ARGs in animal farms upon antibiotic administration, an increase in ARGs has also been observed in the human gut microbiome (Yassour et al., 2016). Secondly, dysbiosis of the gut microbiome has been linked to several other side-effects, such as obesity and a mutated microbial community, which could lead to further complications and need for medical care (Korpela et al., 2016; Shashkova et al., 2016). Thirdly, the gut microbiome is also known to degrade antibiotics which could lead to higher dosage requirements.

Antibiotics are an indispensable part of medical science. The misuse of antibiotics is well known to give rise to MDR pathogens. However, the direct side effects of antibiotics on our health have not been properly addressed. Antibiotics are often administered during the intrapartum period to avoid group B *Streptococcus* (GBS) infection, which is currently one of the major perinatal pathogens, in newborns (Chang et al., 2017; Seale et al., 2017). Although dysbiosis caused by intrapartum antibiotic prophylaxis (IAP) in the newborn gut microbiome appears to recover by 12 weeks (Stearns et al., 2017), higher incidences of ARGs have been reported (Nogacka et al., 2017). Antibiotic treatments in children have also been shown to reduce microbiome diversity with a sharp increase in resistome (Yassour et al., 2016). A study on the oral microbiome of neonates (*n* = 36) showed that it is composed majorly of maternal oral (65.35%) and placental (3.09%) microbiome and holds no resemblance (0%) to the maternal material gut (Gomez-Arango et al., 2017). Furthermore, the oral microbiome was segregated based on maternal exposure to antibiotics during the intrapartum period, indicating that the maternal antibiotics usage has a profound effect on the neonate Furthermore, ARGs carried in mobile elements persisted for a prolonged period. Usage of macrolide in Finnish children (2–7 years old; *n* = 142) was associated with long-lasting dysbiosis in microbial composition, such as reduced Actinobacteria and increased Bacteroidetes, while the change in functional aspects were represented by increased macrolide resistance and reduced bile-salt hydrolase (Korpela et al., 2016).

Antibiotic-induced microbiome depletion (AIMD) is a well-known side effect of antibiotic therapy. AIMD in the gut has been linked to anxiety and depression (Leclercq et al., 2017; Macedo et al., 2017; Pryor et al., 2020). It also causes inflammatory signs, including increased myeloid dendritic cells (mDCs) and plasmacytoid dendritic cells (pDCs) in the blood (Hagan et al., 2019). Microbiome dysbiosis induced by the consumption of macrolide is also associated with increased cases of asthma and a higher risk of obesity (Korpela et al., 2016). AIMD reduces short-chain fatty acids (SCFAs), such as butyrate and serum bile acid (BA), which shifts enterocytes to use glucose as the energy source, which ultimately leads to low serum glucose and a disturbance in glucose homeostasis (Zarrinpar et al., 2018). Studies in murine gut microbiomes have shown than amoxicillin treatment leads to blooms of *Bacteroides thetaiotaomicron*, which upregulate polysaccharide utilization and is further elevated by glucose (Cabral et al., 2019). Antibiotic treatments are also known to cause mutations in the bacterial community (Shashkova et al., 2016).

Recovery of the gut microbiome after antibiotic treatment is an active area of research. Interestingly, dysbiosis of the gut microbiome in humans after antibiotic interventions was found to recover within a week after autologous fecal microbiome transplantation; however, it was prolonged by probiotics which led to changes in taxonomic and functional diversity and lowered bacterial load, leaving the patients susceptible to secondary infections (Suez et al., 2018). Furthermore, in order to protect the microbiota from AIMD, a colon-targeted adsorbent, DAV132, was developed that selectively targets antibiotics and is currently under clinical trial phase 2. Co-administration of DAV132 with moxifloxacin (fluoroquinolones) has been shown to preserve gut microbiota with no adverse effects and also reduce moxifloxacin concentration to 99% in fecal samples (De Gunzburg et al., 2018). Studies of AIMD have shown that the dysbiosis could be predictable based on the initial state of the microbiome. Healthy volunteers (*n* = 18) under cefprozil treatment had similar changes in the microbiome with increased *Lachnoclostridium bolteae* in a majority of participants, despite showing variability on an individual level (Raymond et al., 2016). The authors also noted that a subset of participants, having low diversity of Bacteroides enterotype before the treatment, exhibited enriched *Enterobacter cloacae* after the antibiotic treatments, indicating that the initial status of the microbiome could be a major determinant of the antibiotic therapy outcome.

A majority of the drugs are administered orally, which leaves the drug exposed to various commensals in the gut. Since the human gut microbiota varies at an individual level, the drugs are exposed to unique sets of commensals as per the patient’s microbiome. The human microbiota encodes 150-fold more genes than the human genome. Such a rich reservoir of genes house drug-metabolizing genes that can have various effects on the drugs, such as sulfasalazine activation (Sousa et al., 2014), digoxin inactivation (Haiser et al., 2013), and sorivudine toxification (Okuda et al., 1998). A recent study has shown 271 drugs can be metabolized by 76 human gut bacteria and the drugs with lactones, nitro, azo, and urea groups are more prone to microbial metabolism (Zimmermann et al., 2019). Interestingly, the authors also noted that drug metabolism by the microbiome was significantly correlated to the detection of drug metabolizing gene abundance. Such advances could pave the road to personalized medicine which would provide maximum efficacy and minimal side effects.

## Antibiotics at Sublethal Concentration

Although antibiotics are known to inhibit bacterial growth at MIC or lethal concentration, such concentrations are seldom present in the natural environment. Non-lethal concentrations of antibiotics are also observed in clinical situations, such as during a patient’s non-compliance (Sengupta et al., 2013). It is essential to understand the effects of antibiotics at non-lethal concentrations to shed light on the emergence of ARGs in natural as well as clinical settings. Antibiotics have a diverse role beyond the direct inhibition of competing microbes (Walsh, 2013; Yap, 2013). Global transcriptomic analysis of *Acinetobacter baumannii* under sub-inhibitory concentrations of ciprofloxacin and tetracycline have shown increased ISAba13-mediated mutation, including synonymous and non-synonymous SNP (Fajardo and Martínez, 2008). A multitude of literature has reported on the role of antibiotics as signaling molecules that are involved in quorum sensing, biofilm formation, virulence factors, and host–parasite interaction at sub MIC concentration (Davies, 2009; Anbazhagan et al., 2012). Several antibiotics at sublethal concentrations have been found to induce biofilm formation, indicating that antibiotics could naturally be a signaling molecule (Townsley and Shank, 2017). Recent studies on *Phaeobacter inhibens* treated with broad spectrum tropodithietic acid (TDA) at 100-fold below minimal inhibitory concentration was found to induce similar transcriptomic profiles to that of the quorum sensing (QS) molecule *N*-acyl-homoserine lactone (AHL) (Beyersmann et al., 2017). Biofilm formation requires co-ordination among the bacterial cells to aggregate and form a complex structure. Hence, cell–cell interactions through QS is a mandatory function in biofilm formation. A similar effect of TDA to that of QS molecule AHL could indicate that TDA might serve as a signaling molecule in natural settings. Another interesting example is the role of antibiotic prodiginines in *Streptomyces coelicolor*. The filamentous *Streptomycetes* reproduce by sporulation and formation of mycelium. During reproduction, older mycelium is consumed as a substrate. Prodiginines are accumulated internally during mycelium differentiation that could serve as programmed cell death and also as a chemical protection of the nutrients from competing microbes (Tenconi et al., 2018). Such studies provide clear evidence that natural antibiotics have a diverse set of roles. Antibiotics at concentrations below MIC have also been found to be beneficial to the susceptible bacteria (Linares et al., 2006). Sublethal concentrations of antibiotics have also been shown to accelerate the emergence of ARGs, and increase mutation rates and horizontal gene transfer (Andersson and Hughes, 2014; Gullberg et al., 2014; Ben et al., 2019). Furthermore, minimal concentrations of antibiotics in WWTP have also been shown to enrich ARGs (Lundström et al., 2016).

## Conclusion

Although resistance to antibiotics has made the use of traditional antibiotics obsolete, combinatorial antibiotic therapy together with nanoparticles and phytochemicals could be an effective alternative solution for antibiotic resistance. Since resistance to antibiotics and their alternatives are an inevitable part of microbial evolution, surveillance of ARGs should be considered the first priority in future policy making. Whole genome analysis and metagenomics have made remarkable advancements with the aid of NGS technology and bioinformatics. The results of such techniques are quite reliable and their implementation in routine diagnostic laboratories should be encouraged. In addition to the negative impact of antibiotics’ over-usage, it should be noted that adequate application of antibiotics itself could cause major dysbiosis in the gut microbiome, which ultimately could lead to several side effects.

## Author Contributions

RK, MI, DB, ST, PG, and VA conceptualized the review. RK, MI, and JM wrote the manuscript. All the authors read and approved the final manuscript.

## Conflict of Interest

The authors declare that the research was conducted in the absence of any commercial or financial relationships that could be construed as a potential conflict of interest.
